# *Salmonella enterica* Serovar Typhimurium Lacking *hfq* Gene Confers Protective Immunity against Murine Typhoid

**DOI:** 10.1371/journal.pone.0016667

**Published:** 2011-02-09

**Authors:** Uday Shankar Allam, M. Gopala Krishna, Amit Lahiri, Omana Joy, Dipshikha Chakravortty

**Affiliations:** Department of Microbiology and Cell Biology, Centre for Infectious Disease Research and Biosafety Laboratories, Indian Institute of Science, Bangalore, India; University of Hyderabad, India

## Abstract

*Salmonella enterica* is an important enteric pathogen and its various serovars are involved in causing both systemic and intestinal diseases in humans and domestic animals. The emergence of multidrug-resistant strains of *Salmonella* leading to increased morbidity and mortality has further complicated its management. Live attenuated vaccines have been proven superior over killed or subunit vaccines due to their ability to induce protective immunity. Of the various strategies used for the generation of live attenuated vaccine strains, focus has gradually shifted towards manipulation of virulence regulator genes. Hfq is a RNA chaperon which mediates the binding of small RNAs to the mRNA and assists in post-transcriptional gene regulation in bacteria. In this study, we evaluated the efficacy of the *Salmonella* Typhimurium Δ*hfq* strain as a candidate for live oral vaccine in murine model of typhoid fever. *Salmonella hfq* deletion mutant is highly attenuated in cell culture and animal model implying a significant role of Hfq in bacterial virulence. Oral immunization with the *Salmonella hfq* deletion mutant efficiently protects mice against subsequent oral challenge with virulent strain of *Salmonella* Typhimurium. Moreover, protection was induced upon both multiple as well as single dose of immunizations. The vaccine strain appears to be safe for use in pregnant mice and the protection is mediated by the increase in the number of CD4^+^ T lymphocytes upon vaccination. The levels of serum IgG and secretory-IgA in intestinal washes specific to lipopolysaccharide and outer membrane protein were significantly increased upon vaccination. Furthermore, *hfq* deletion mutant showed enhanced antigen presentation by dendritic cells compared to the wild type strain. Taken together, the studies in murine immunization model suggest that the *Salmonella hfq* deletion mutant can be a novel live oral vaccine candidate.

## Introduction

*Salmonella*e are Gram-negative, facultative intracellular pathogens, causing variety of diseases in multiple hosts with different disease outcome. The genus *Salmonella* is composed of two distinct species: *Salmonella bongori*, a commensal of cold-blooded animals and *Salmonella enterica*. *Salmonella enterica* has 6 subspecies, and each subspecies have associated serovars that differ by antigenic specificity and comprises of more than 2500 serovars [1]. Serotypes within subspecies I (*S. enterica* subsp. *enterica*) are responsible for the vast majority of the *Salmonella* infections in warm-blooded animals [2]. These serotypes differ widely in a variety of features, most notably in their host range, severity and type of disease they typically cause. *S*. *enterica* serovar Typhimurium is an important cause of food poisoning and human gastroenteritis and has added significance as a model of human typhoid fever in mice. *S.*Typhimurium infection is often fatal if remained untreated in immuno-compromised patients [2], [3].

*S. enterica* serovar Typhi (*S.*Typhi) and *S. enterica* serovar Paratyphi (*S*.Paratyphi) cause typhoid and paratyphoid fever in humans; mostly in developing and under developed countries. They still remain a significant public health problem in many parts of the world. Annual global burden of typhoid is about 21.2 million cases and death rate of ∼2.2 lakh individuals per year worldwide, whereas paratyphoid accounts to 5.4 million cases per year globally [4], [5]. WHO reports that in some developing countries of Asia and Africa, the annual incidence of typhoid fever may reach 1% with case fatality rates as high as 10% [6].

Vaccination has been practiced for many years and it is one of the most effective methods of controlling infectious diseases like typhoid. Currently two licensed vaccines against *Salmonella* are in use globally, Vi polysaccharide vaccine and Ty21a live attenuated vaccine [7]. Typhim Vi is the purified Vi antigen from *S.* Typhi and vaccination with Typhim Vi provides 55–75% protection in one single dose [8]. The other licensed typhoid vaccine is Vivotif Berna which is an attenuated live *Salmonella* Typhi Ty21a strain, generated by chemical mutagenesis of parental virulent strain of *Salmonella* Typhi Ty21 [9]. Vivotif is highly immunogenic, but an important practical shortcoming is its three to four dosage requirement of 2–6×10^9^ CFU every alternate day [10]. Most of the *Salmonella* based vaccines generated till date has been created by deleting essential genes of the metabolic pathway, pathogenicity islands or global regulators. *Salmonella* harboring mutations in SPI-2 [11], aroA [12], [13], hrtA [14], phoP [15], rpoS [16], rfaH [17], dam [18], [19], [20], trxA [21], FoF_1_ ATPase [22] have been tested as vaccine candidates in animal models. The major challenge in the development of live attenuated *Salmonella* vaccines is generating a safe yet immunogenic vaccine strain.

Hfq is a RNA chaperon which plays a vital role in regulating the stability as well as the translational activity of several mRNAs through small non-coding RNA. Initially, Hfq was identified in *E. coli* as a host factor essential for replication of bacteriophage Qβ [23]. Later it was found that the *E.coli hfq* deletion mutant exhibits pleiotropic phenotypes that include decreased growth rate, change in the morphology, altered sensitivity to UV light and oxidants [24]. It has now been shown that Hfq impedes the translation of the major stress sigma factor, σ^S^ (RpoS) in the enteric bacteria viz *E.coli* and *Salmonella* [25], [26]. Hfq has also emerged as a key player in mRNA translational control by small non-coding RNAs (sRNAs) [27]. Studies later demonstrated that Hfq was involved in *Salmonella* pathogenesis through its effect on expression and secretion of virulence factors (in a σ^S^-independent manner) resulting in an attenuation of the *Salmonella hfq* deletion mutant in cell culture system as well as in murine model [28]. Global microarray and proteomic analyses of *Salmonella* Typhimurium grown aboard the space shuttle mission STS-115 identified Hfq as the global regulator involved in the response to the space flight environment as well [29]. Therefore, it becomes evident that Hfq is a bacterial RNA binding protein having many important physiological roles.

The aim of the present study is to evaluate the efficacy of *Salmonella enterica* serovar Typhimurium lacking *hfq* gene as a candidate for live oral vaccine against *Salmonella* infection in murine salmonellosis model. The data suggests that the STMΔ*hfq* is able to elicit protective immune responses against oral challenge with *Salmonella* virulent strain. Further, the vaccination with STMΔ*hfq* is safe in the pregnant mice. The protective response elicited by the mutant strain is characterized by an increase in total CD4^+^ T lymphocytes population, serum IgG and intestinal secretary-IgA antibodies.

## Results

### *Salmonella hfq* deletion mutant construction and *in vitro* characterization

**Figure 1:**
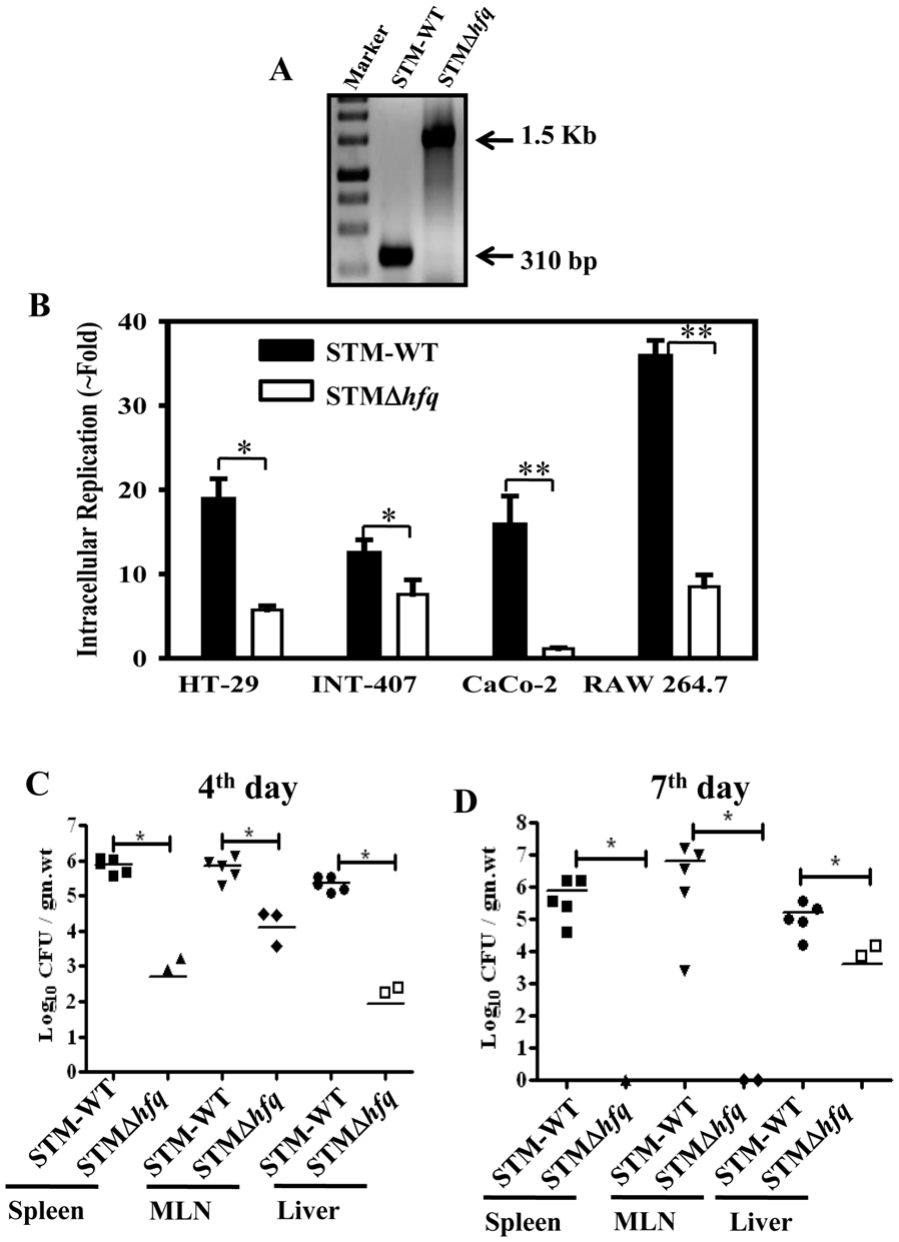
STMΔ*hfq* is highly attenuated *in vitro* and *in vivo* (A) Generation of STMΔ*hfq* by one step gene inactivation. Confirmatory PCR showing the presence of Kanamycin cassette (1.5 Kb) in STMΔ*hfq*. (B) Intracellular replication of STM-WT and STMΔ*hfq* strains in INT-407, HT-29, CaCo-2 and RAW 264.7 cell lines. Cells were infected with the STMΔ*hfq* or STM-WT at an MOI of 10. Data shows fold increase from 2 h to 16 h. All the experiments were done in triplicate. (C&D) Organ loads of STM-WT and STMΔ*hfq*. Two groups of mice (5 each) were infected with 10^7^ CFU/mouse orally and sacrificed on day 4 (C) and day 7 (D) of post infection. Bacterial counts in spleen, MLN and liver were shown as CFU/gm.wt with standard errors. Plots are representative of two independent experiments with similar results. Statistical significance was defined as follows: (*p<0.05; ** p<0.005) (Student's *t* test & Mann-Whitney U test).

*hfq* gene was deleted from *Salmonella enterica* serovar Typhimurium using the lambda red recombination system [30] and mutants were confirmed by colony PCR with confirmatory primers against the sequence flanking the *hfq* gene (Figure 1A). The same were also confirmed using primers against the kanamycin cassette (data not shown). We then compared the growth of the *hfq* deletion mutant with that of the STM-WT in rich media (LB) and defined media (minimal media). While the number of viable bacteria was found to be same for equal OD of both the cultures, the *hfq* mutant reached stationary phase at later time than STM-WT in both LB as well as in the minimal media (data not shown).

*Salmonella* encounters two major cells during its course of pathogenesis namely epithelial cells and macrophage cells which are the main cells that support the bacterial growth *in vivo* [31]. Infection of cultured phagocytic and epithelial cells mimics relevant *in vivo* host-pathogen interactions, bacterial invasion and intracellular multiplication. Therefore, we checked the intracellular replication capacity of the *hfq* mutant in human epithelial as well as murine macrophage cell lines. The *hfq* mutant is highly defective in its replication capacity (3-5 fold) as compared to that of the WT in all the cell lines tested except CaCo-2. In CaCo-2 cells, the mutant is highly attenuated and the fold replication difference is ∼15-fold (Figure 1B). This implies that the *hfq* mutant is attenuated in both the macrophage and epithelial cell lines (Figure 1B).

### Attenuation of the *Salmonella hfq* deletion mutant in mouse model

Having observed the attenuation of STMΔ*hfq* in epithelial as well as in macrophage cell lines, we decided to investigate the virulence of the mutant strain in the murine model of typhoid fever. To analyze the bacterial colonization in different organs, group of BALB/c mice were infected orally with 10^7^ CFU of WT *Salmonella* and STMΔ*hfq*. As shown in Figure 1C and D, organ load of the STMΔ*hfq* was significantly lower (≈100-1000 fold, p<0.05) when compared to the STM-WT, 4^th^ day post infection in spleen, liver and mesenteric lymph nodes. The STMΔ*hfq* was completely cleared by 7^th^ day post infection unlike the STM-WT. This suggested that the mutant is severely attenuated and is efficiently cleared from the different organs of mice.

### Complementation of *hfq* gene restored STMΔ*hfq* virulence

The wild type *hfq* gene was cloned into pQE60 vector and the resultant pQE60*hfq* plasmid was transformed into STMΔ*hfq* strain. The WT, mutant and STMΔ*hfq*pQE60*hfq* were used for complementation studies in cell lines as well as in murine model. The complemented strain behaved like the WT in terms of intracellular replication in both RAW 264.7 and INT-407 cell lines as well as in animal model (Figure S1 A&B). The bacterial burden in spleen, liver and mesenteric lymph nodes at single time point (4^th^ day) suggests that the tans-complementation with *hfq* gene restored the mutant's virulence (Figure S1 C).

### Safety of the *Salmonella hfq* deletion mutant

To determine the extent of the effect of the *hfq* deletion on *Salmonella* virulence, we determined LD_50_ values using the STMΔ*hfq* in female BALB/c mice. The LD_50_ value of the wild-type was 4×10^4^ CFU for oral administration. In case of mice infected with STMΔ*hfq*, LD_50_ could not be estimated because mice did not die even after oral inoculation with the maximum dose of 10^9^ CFU/mouse. The mice that survived infection with the STMΔ*hfq* did not show any signs of illness and remained healthy for the entire duration of the experiment.

**Figure 2:**
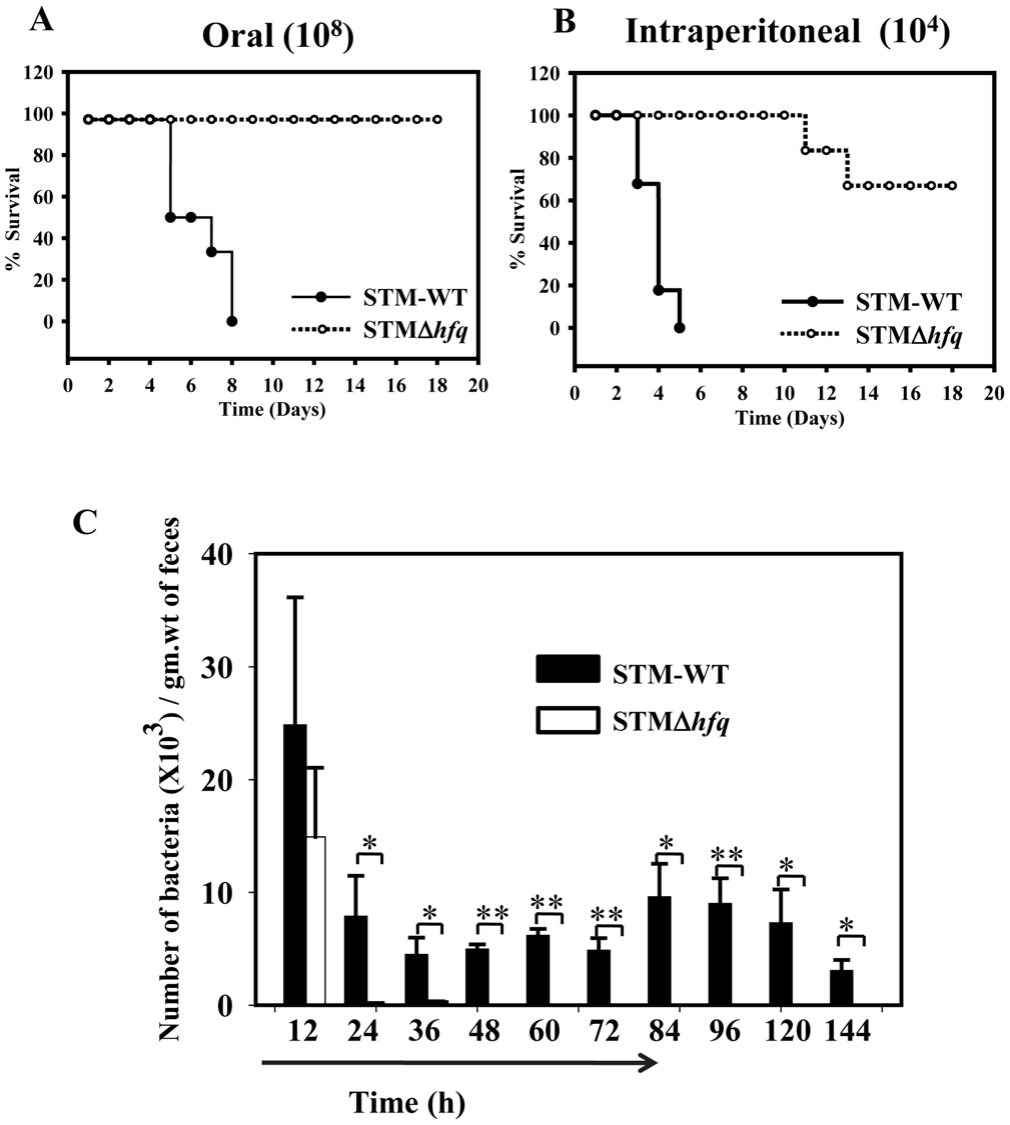
STMΔ*hfq* mutant is highly safe for infection in murine model. (A & B) Survival curves. Mice were infected orally (A) at a dose of 10^8^ or intraperitoneally (B) with a dose of 10^4^ bacteria per mouse (n = 10) and mice were observed twice daily for survival. (C) Reduced fecal shedding of the STMΔ*hfq*. Mice were infected orally with the STM-WT or STMΔ*hfq* mutant strains at a dose of 10^7^ bacteria/mouse. Uninfected mice were used as control. Fresh fecal pellets were collected at the indicated time points and shedding was determined by plating on culture media. Data is represented as the number of bacteria per gram weight of the fresh fecal pellets. Shown are the combined results from two experiments with 5 mice in each group. Statistical significance was defined as follows: (*p<0.05) (Student's *t* test & Fischer's exact test).

Next, we studied the survival of mice, upon infection with the STM *hfq* deletion mutant. The infection was carried out by either oral (10^8^) or intraperitoneal route (10^4^) and the survival was monitored for 3 weeks. Infection with the wild type strain through either route led to an early onset of symptoms in the mice followed by death within ten days, whereas 100% percent survival was observed in case of mice infected with STMΔ*hfq* through oral route. However, in intra-peritoneal infection, the percent survival of mice was found to be 70% in the case of STMΔ*hfq.* (Figure 2A&B). The surviving mice remained healthy up to 7 weeks (monitored for 7 weeks only). The high attenuation of the STMΔ*hfq* mutant given through oral route thus renders it highly safe for infection in mice.

The high levels of shedding exhibited by *Salmonella* Typhimurium strains have precluded their development as live *Salmonella* vaccines. Therefore, we compared the fecal shedding of the STM-WT and the vaccine strain STMΔ*hfq* in the murine model. As shown in Figure 2C, the vaccine strain was shed lesser in the feces as compared to its wild type counterpart. Shedding of the vaccine strain was observed up to 36 h of post administration and no *Salmonella* were detected exceeding the time period. No *Salmonellae* were recovered from the fecal matter of naive mice. Our results conclusively demonstrate that the STMΔ*hfq* vaccine strain is shed lesser in the feces.

### STMΔ*hfq* imparts protection against subsequent challenge with the virulent *Salmonella* in mice

**Figure 3:**
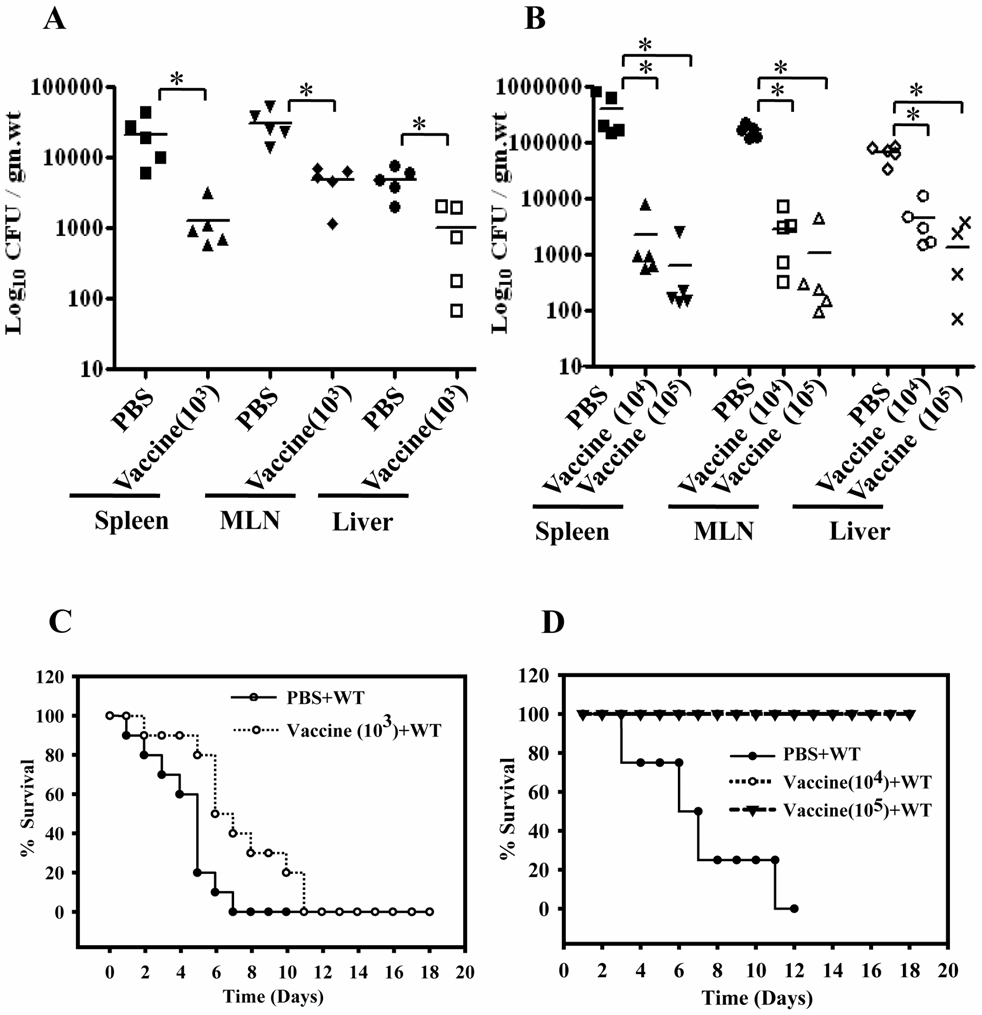
Multiple immunizations with STMΔ*hfq* confer protection against virulent *Salmonella*. Groups of 5 mice were immunized orally with 10^3^ (A&C), 10^4^ or 10^5^ (B&D) STMΔ*hfq* for two booster doses followed by challenge with WT through oral route (for organ load: 10^7^ and for survival analysis: 10^8^ of WT) 7 days after the final booster dose. Control mice received PBS. 7 days after challenge, mice were sacrificed and bacterial load in different organs was enumerated using direct cultures of serially diluted homogenized samples. A&B depict bacterial load in different organs and C&D shows survival curves. Result presented is one of two independent experiments performed. Statistical significance was defined as follows * p<0.05; ** p<0.005 (Mann-Whitney U test & Fischer's exact test).

A good vaccine candidate should be attenuated, safe, immunogenic and should give protection against a lethal challenge with minimum dose of priming. Having demonstrated that the vaccine strain is attenuated and safe in the murine *Salmonella* model, next we examined the ability of attenuated STMΔ*hfq* strain to generate protective immunity against the virulent *Salmonella* infection. We followed the immunization strategy as given in Figure S2. Cohorts of five mice each were orally primed with the STMΔ*hfq* at a dose of 10^3^, 10^4^ or 10^5^ bacteria/mouse followed by two booster doses (day 7 and 14). Control mice received PBS. All the subsequent steps (challenge, sacrifice) were carried out with a gap of seven days between them. Seven days after the final booster dose, the mice were challenged with 10^7^ CFU of STM-WT through oral route for analyzing organ infiltration. For survival assay, mice were challenged with 10^8^ CFU/mouse through oral route. In case of mice immunized with 10^3^ STMΔ*hfq* and boosted twice with the same dose, the mice showed a significant reduction (*P* = 0.0079) in the bacterial load upon challenge compared to the organs of the control unprimed mice (Figure 3A). However, all the vaccinated mice did succumb to death upon challenge with the STM-WT like the placebo control mice within 10 days (Figure 3B). We then increased both the oral immunization and the booster doses to 10^4^ and 10^5^ CFU/mouse. In case of the mice primed with either 10^4^ or 10^5^ vaccine strain, there was a substantial decrease (*P* = 0.0079) in the bacterial burden and none of the vaccinated mice succumbed to death upon challenge with STM-WT (Figure 3C & D). Therefore, the *hfq* deletion mutant gives protection against subsequent challenge with the virulent *Salmonella* strain.

**Figure 4:**
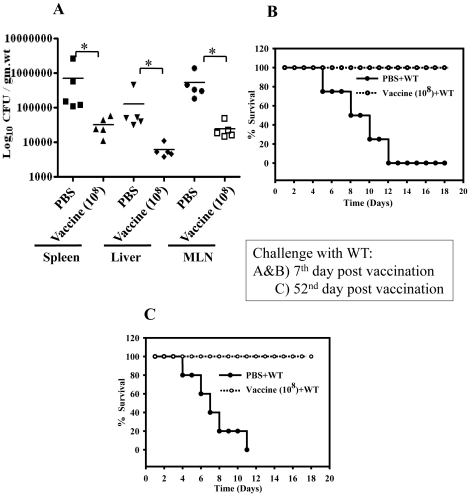
Single dose of vaccination imparts protection against WT *Salmonella* and induces long-term immunological memory. BALB/c mice were vaccinated through oral route with 10^8^ CFU of STMΔ*hfq* or PBS and challenged with either 10^7^ CFU of WT for assessing organ load (A) or 10^8^ of WT for assessing mortality (B). 7 days after challenge, mice were sacrificed and bacterial load was enumerated. For long-term immunological memory mice were challenged orally after 52 days of vaccination (C). Each group contains 5-6 mice. Statistical significance was defined as follows * p<0.05 (Mann-Whitney U test & Fischer's exact test).

Another important factor determining efficiency of the live attenuated oral vaccine is the number of doses. So, we further evaluated whether a single dose of the STMΔ*hfq* priming is sufficient to give protection against challenge with virulent *Salmonella*. A cohort of five mice were vaccinated with 10^8^ CFU of STMΔ*hfq* and challenged with 10^7^ WT bacteria per mouse on 7^th^ day post vaccination. We observed that there was an average of 15-fold reduction (*P* = 0.0079) in the organ load of mice with single dose vaccination (Figure 4A). Moreover as shown in Figure 4B, single dose of vaccination followed by challenge after 7 days with the STM-WT (10^8^) resulted in 100% survival whereas unvaccinated mice succumbed to death within two weeks. This data strongly suggests that single dose of vaccination with the STMΔ*hfq* can confer protection against the virulent *Salmonella*.

### Long term immunological memory induction by the STMΔ*hfq* vaccine strain

One of the problems with oral live attenuated vaccine has been the lack of long-term immunological memory. Thus, we examined the capacity of mice vaccinated with single dose (10^8^) to mount a recall response 52 days post vaccination (45 days after clearance). Age matched unvaccinated challenged mice died within two weeks after challenge whereas vaccinated challenged mice showed 100% survival (Figure 4C). This data indicates that the STMΔ*hfq* is able to induce long-term immunological memory.

### STMΔ*hfq* vaccine strain is safe in pregnant mice

Pregnancy is a transient immuno-compromised condition and leads to increased susceptibility to invading pathogens, resulting in abortion and congenital defects in the fetus [32], [33], [34], [35]. Thus, the possibility of pregnancy failure after challenge with the STMΔ*hfq* and WT in pregnant mice model was studied. Two groups of ten mice were administered orally with STM-WT and STMΔ*hfq* vaccine strain at 12–15 days of gestation (mid pregnancy stage) with a dose of 10^8^. Mice were scored for the delivery of pups and their survival every day post infection. We found that the STM-WT virulent strain caused the death of four mice and induced abortion in three others. Only three mice delivered pups normally; but 14 out of 16 pups died within 48 h after delivery. On the other hand, out of 10 pregnant mice administered with the STMΔ*hfq* mutant strain, one mouse died but remaining mice delivered normal healthy pups and the pups remained healthy up to 7 weeks (monitored for 7 weeks only, Table 1).

**Table 1:** Safety of the STMΔ*hfq* vaccine strain in pregnant mice.

No. of pregnant mice	Relevant genotype	No. mice died before delivery (healthy/dead)	No. of abortions	Death of pups after delivery (no. of death/total no.)
10	STM-WT	6/4	3	14/16
10	STMΔ*hfq*	9/1	0	0/32

Pregnant BALB/c mice (12–15 days of gestation) were infected orally at a dose of 10^8^ bacteria per mouse with the STM-WT or the vaccine strain STMΔ*hfq* and observed for abortion and the offspring outcome.

### Oral vaccination with the STMΔ*hfq* increases the total number of splenic CD4^+^ T cells

**Figure 5:**
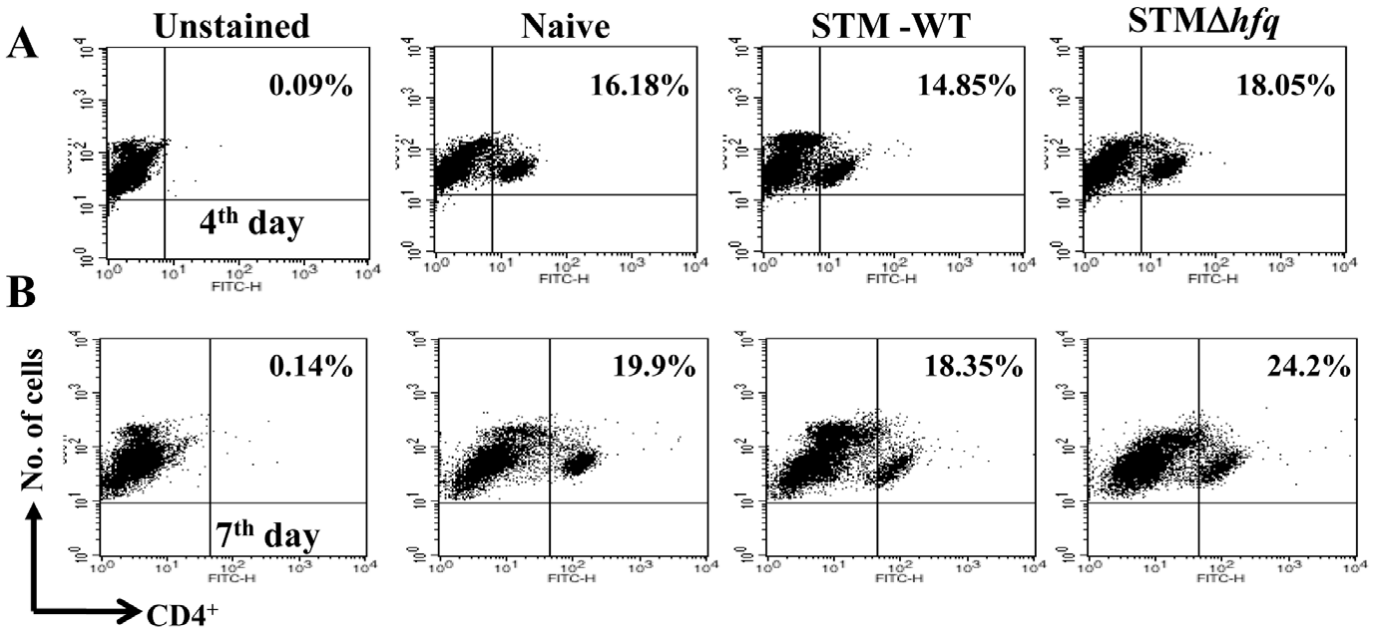
Flow cytometric analysis of CD4^+^ T cell population in the spleen on 4^th^ day (A) and 7^th^ day (B) post infection. Group of mice were inoculated with the STM-WT or STMΔ*hfq* with dose of 10^7^ bacteria per mouse. Uninfected mice were used as control. Splenocytes were isolated on 4^th^ and 7^th^ day post infection from both infected and control mice and stained with FITC-conjugated anti-CD4 MAb. The relative levels of CD4^+^ T-lymphocytes were measured through FACS. Data was analyzed with BD Cell-Quest software and represented by dot plot. The results are representative of two independent experiments. Each group consisted of 4-5 mice. Statistical significance was defined as follows: (* p<0.05) (Student's *t* test).

To address the mechanism responsible for the protective immunity conferred by the vaccine strain, we studied the status of CD4^+^ and CD8^+^ T-lymphocyte population in the orally infected mice (10^7^). Total splenocytes were isolated from the naive mice and mice infected with either STM-WT or STMΔ*hfq* at various time points (4^th^ and 7^th^ day post infection) and stained with anti-CD4^+^ or anti-CD8^+^ specific antibodies. Although no significant difference was observed in the CD4^+^ T-lymphocyte population in the vaccinated mice at 4^th^ day post infection, there was a≈6% increase (*P* = 0.003) in the same at 7^th^ day post infection when compared to that in the STM-WT infected mice (Figure 5A & B).

**Figure 6:**
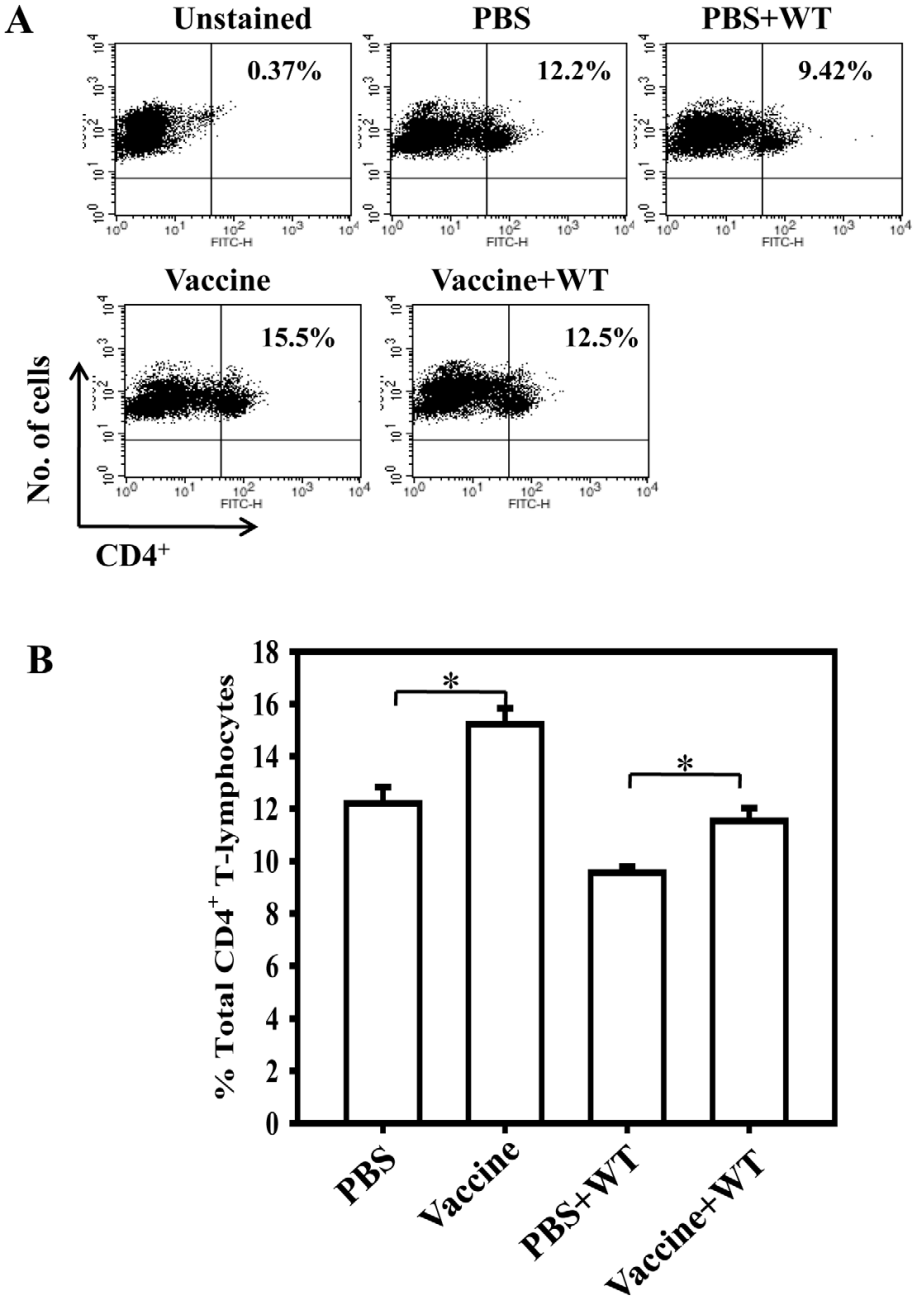
Analysis of splenic CD4^+^ T cell population in vaccinated and unvaccinated mice with or without challenge. Group of mice were orally given PBS or 10^8^ STM Δ*hfq* bacteria and then challenged with 10^7^ CFU of the STM-WT per mouse. On 7^th^ day post vaccination and 7^th^ day post challenge, single cell suspension of splenocytes were prepared and stained with FITC-conjugated anti-CD4 MAb. The relative levels of CD4^+^ T-lymphocytes were measured through FACS. Data was analysed by BD Cell-Quest software and represented through dot plot (A) and bar graph (B). Statistical significance was defined as follows: (* p<0.05) (Student's *t* test). Each group consisted of 4-5 mice.

Next, we determined whether the oral vaccination with STMΔ*hfq* (10^8^) affects the status of CD4^+^ and CD8^+^ T-lymphocyte population with or without WT challenge (10^7^). Total splenocytes were isolated from naive and vaccinated mice (at 7^th^ day post vaccination) and from unvaccinated–challenged and vaccinated-challenged mice (at 7^th^ day post challenge). The mice vaccinated with the STMΔ*hfq* showed significant increase (*P* = 0.0129) in the helper T cell population compared to that in the unvaccinated mice. The level of helper T cells was considerably higher in the vaccinated-challenged mice in comparison to that in the PBS-challenged ones (*P* = 0.012) (Figure 6A &B). However, there was no change in the number of CD8^+^ T cells in both the cases (Figure S3&S4). Interestingly, we observed that the numbers of CD4^+^ T cells were slightly reduced with the STM-WT infection. Thus our results suggest that vaccination induces CD4^+^ T-cell population and maintains the same even after infection, without altering the CD8^+^ T-lymphocyte levels.

### Vaccination with the STMΔ*hfq* increases the serum IFN-γ and IL-6 levels

**Figure 7:**
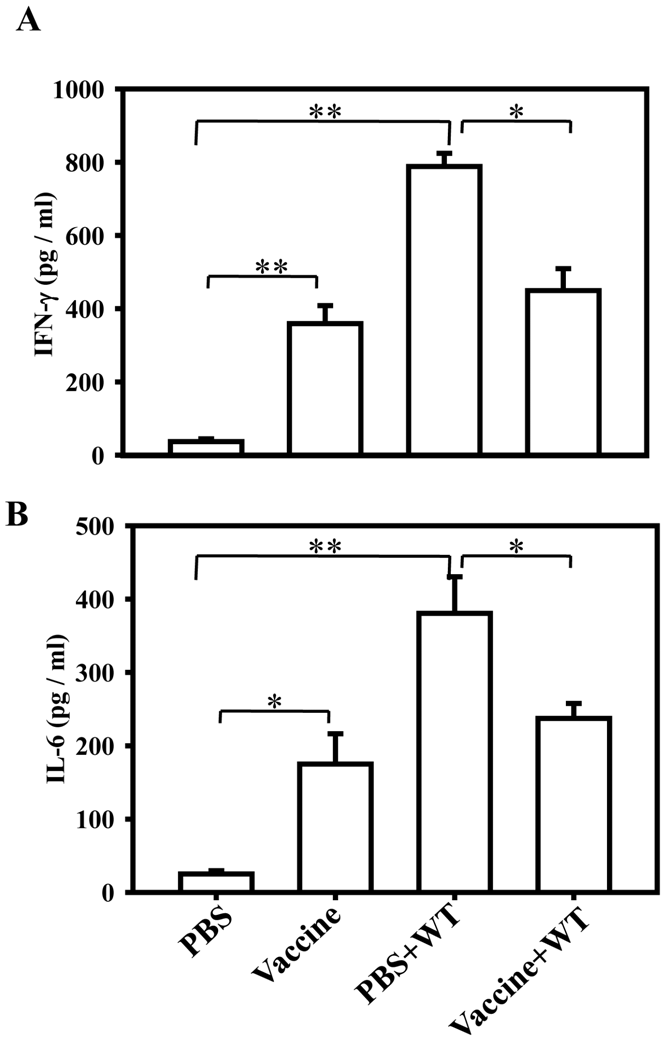
Analysis of serum cytokine levels with single dose of vaccination. (A) IFN-γ (B) IL-6. Group of mice were orally given PBS or STMΔ*hfq* (10^8^) and then challenged with STM-WT (10^7^ CFU/mouse) at 7^th^ post vaccination. Serum was collected on 7^th^ day post vaccination (from unchallenged mice) and 7^th^ day post challenge (from challenged mice) and cytokine levels were measured using ELISA. Data is representative of two independent experiments (n = 4-5). Statistical significance was defined as follows: (*p<0.05; ** p<0.005) (Student's *t* test).

Cytokine profile of the serum after vaccination indicates the kind of immune response elicited. Th1 cytokines (IFN-γ, IL-2 and TNF-α) are important for cell mediated immunity, while the Th2 cytokines (IL-4, IL-6 and IL-10) are important for increased antibody production [36]. Serum levels of IFN-γ and IL-6 in vaccinated (10^8^) and PBS administered mice, with or without challenge (10^7^) were estimated. Vaccinated mice had higher serum IFN-γ and IL-6 than the PBS administered mice (*P* = 0.0029) The serum IL-6 and IFN-γ levels of vaccinated mice further increased upon challenge while remaining lower than that of unvaccinated challenged mice (Figure 7A & B). In summary, our data suggests that the oral vaccination with the STMΔ*hfq* increases the IFN-γ and IL-6 levels in the serum and might help the host indirectly to reduce the count of virulent *Salmonella*.

### Vaccination with STMΔ*hfq* markedly elicits the humoral immune response

**Figure 8:**
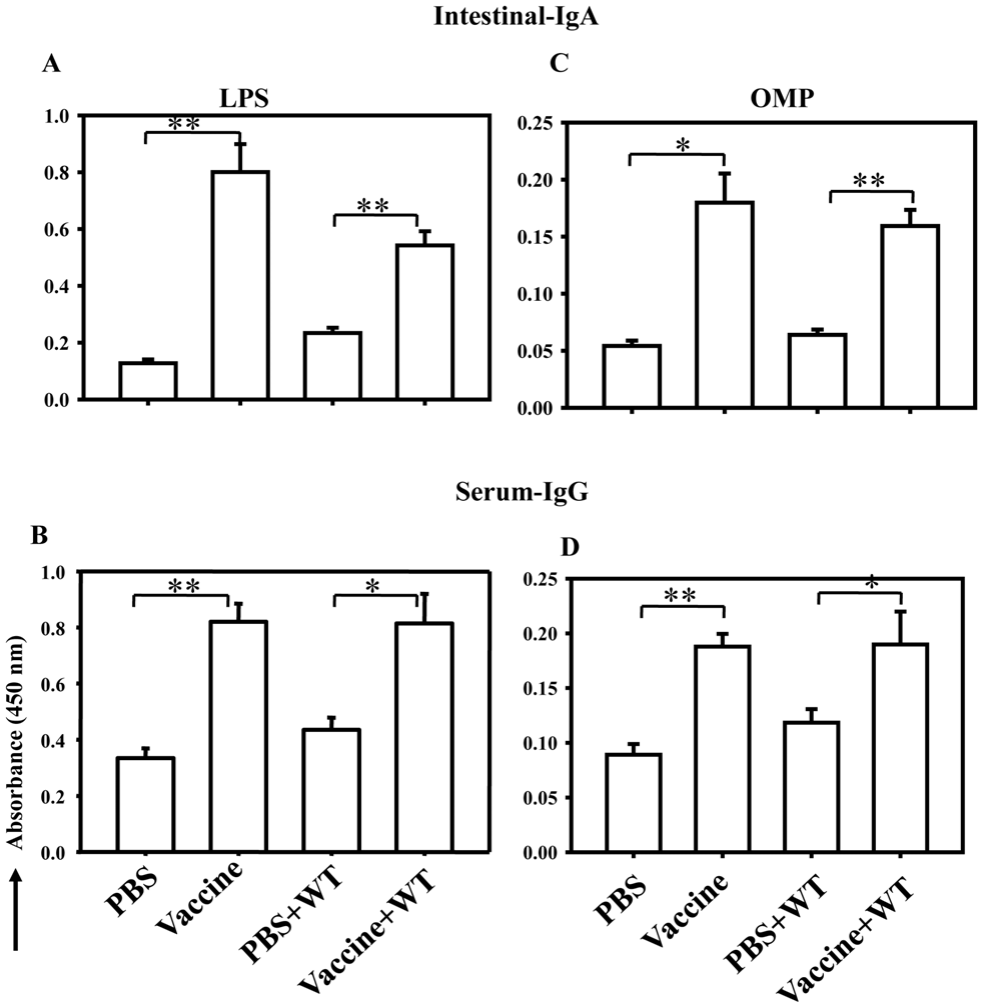
Humoral immune response elicited by single dose of vaccination with STMΔ*hfq*. Group of mice were orally given PBS or STMΔ*hfq* (10^8^) and then challenged with STM-WT (10^7^ CFU/mouse) at day 7 of post vaccination. Serum and intestinal mucus were collected on 7^th^ day post vaccination (from unchallenged mice) and 7^th^ day post challenge (from challenged mice). Serum IgG (B&D) and intestinal S-IgA (A&C) antibodies specific for LPS and OMP were measured by ELISA. The samples were assayed in triplicate and the antibody titer is expressed as the absorbance at 450 nm. Data is representative of two independent experiments. Statistical significance was defined as follows: (*p<0.05; ** p<0.005) (Student's *t* test). (n = 4-5).

Protection against *Salmonella* is mediated through mucosal and serum antibodies (Secretory IgA and serum IgG) as well as cell mediated immunity [37]. The increase in secretory IgA as well as serum IgG antibodies are surrogate markers of protection conferred by an efficient oral vaccine candidate [38]. In order to check whether vaccination with the STMΔ*hfq* increased antibody titers, we measured mucosal as well as serum antibody levels in the vaccinated (10^8^) versus control mice with and without challenge (10^7^). We determined the antibody levels against two surface antigens of the *Salmonella*, namely LPS (Figure 8A & B) and outer membrane proteins (OMPs) (Figure 8C & D). Mucosal IgA and serum IgG levels markedly increased in vaccinated mice compared to naive mice (*P*<0.05). The antibody titers remained high in case of mice vaccinated and challenged compared with the unvaccinated and challenged mice (*P*<0.05). The elicited humoral immune response remained 4 weeks after immunization with mutant strain (Figure S5). It is evident from our results that STMΔ*hfq* induces an effective humoral immune response.

### STMΔ*hfq* is presented better than the STM-WT in an *in vitro* antigen presentation assay

**Figure 9:**
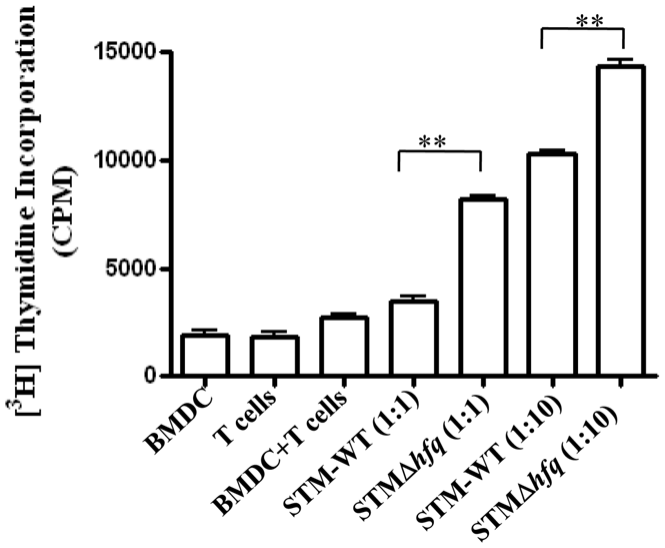
Increased antigen presentation of the STMΔ*hfq* by Denditic cells. Dendritic cells were isolated from the BALB/C mice and were plated at 5×10^4^ cells/well in 96 well flat-bottomed tissue culture plates. C57BL/6 mice were infected with STM-WT for pre stimulation and T-lymphocytes were isolated on 5^th^ day aseptically. DCs were infected with the STM-WT and STMΔ*hfq*. The infected DCs were then co-cultured with T-lymphocytes at a ratio of (1∶1) and (1∶10) for 72 h and then pulsed for 16 h with [^3^H] thymidine (1 µCi/well). The incorporation of [^3^H] thymidine in proliferating T cells were measured by liquid scintillation counter. T cell proliferation was represented counts/min using a beta-counter. The results are representative of two independent experiments in triplcate. Statistical significance was defined as follows: (*p<0.05) (Student's *t* test).

Hopkins et al. in a previous study had reported that the dendritic cells internalize *Salmonella* early after oro-gastric inoculation of mice or injection in a ligated loop of intestine [39]. Dendritic cells are principle components of the innate immune system which are able to prime adaptive immune response by acting as antigen presenting cells [40]. Dendritic cells capture or sample antigens in the peripheral tissue, transport antigens to the lymph nodes and present the processed antigens to T cells via the major histocompatibility complex (MHC) molecules [41]. Thus in order to understand the immunogenicity of the STMΔ*hfq* vaccine strain we compared the stimulation of T cell proliferation by bone marrow-derived dendritic cells (BM-DC) which are infected with equal number of STM-WT or STMΔ*hfq* in an *in vitro* T cell proliferation assay (number of viable cells are same upon infection with WT and mutant). STMΔ*hfq* infected DCs stimulated the T cell proliferation to considerably (*P* = <0.005) higher levels than the STM-WT infected DCs. The lymphocyte proliferation is further enhanced with increased ratio of DC to T cells (Figure 9). Further the viability of DCs infected with WT or STMΔ*hfq* remains unchanged (Data not shown). This clearly demonstrates that the vaccine strain STMΔ*hfq* is presented better than the STM-WT.

## Discussion

In this study, we evaluated the efficacy of the highly attenuated STMΔ*hfq* mutant to serve as a live vaccine against salmonellosis in the murine typhoid model. We have constructed hfq deficient strain of *S.* Typhimurium and investigated the virulence of this strain. The deletion mutant was compromised in its intracellular proliferation capacity and was also severely attenuated *in vivo* following oral infection of BALB/c mice. Trans-complementation of *hfq* gene restored virulence in the mutant. These results are in accordance with the work of Sittka et al. [28] where they reported that the *S.* Typhimurium *hfq* mutant was attenuated in competition with the parental strain in an intraperitoneal infection of BALB/c mice and that the bacterial recovery was also low after oral infection.

Hfq is believed to directly or indirectly regulate at least 20% of all *Salmonella* genes indicating that Hfq acts as a true global regulator in *Salmonella* [42]. Hfq predominantly affects the expression of two type three secretion systems (T3SS) encoded by *Salmonella* pathogenicity islands (SPI), namely SPI-1 and SPI-2. *Salmonella* uses these type three secretion systems (T3SS) to deliver effectors into host cells thereby facilitating host invasion and intracellular survival [27], [28], [42]. The genes regulated by Hfq also include many LPS biosynthesis related genes and ABC transporters [43], [44] which are very important in imparting resistance to antimicrobial peptides. Hfq also regulates iron uptake and storage in bacteria through small RNAs. The available literature shows Hfq's multi-functional role in *Salmonella* biology. Therefore, the *hfq* deletion mutant was evaluated for its efficacy as a potential vaccine strain.

Our data clearly suggests that the STMΔ*hfq* mutant is an effective oral vaccine candidate in the murine model and can be given both in booster as well as in single dose. Vaccination with subsequent challenge reduced the bacterial burden significantly as compared to the unvaccinated mice. Moreover, the bacterial load was completely cleared within 3 weeks post challenge in vaccinated mice (data not shown). Lower vaccine dose (10^3^, given twice in boosters) however, was incapable of rescuing the mice from a lethal WT challenge due to insufficient priming. Hence when the immunization dose was raised to 10^4^ or 10^5^, the mice showed 100% survival. Furthermore, the STMΔ*hfq* also conferred long term protection against challenge. This suggests that the vaccine strain is probably effective in inducing a specific adaptive immune response, resulting in long term protection against *Salmonella* infection.

One of the major pitfalls of live attenuated oral vaccine strains is environmental contamination through fecal matter, which in turn is associated with health and safety concerns. Studies with human volunteer trials of different *Salmonella* Typhimurium vaccine strains showed greater shedding of the vaccine strains in the feces [45], [46], [47]. The degree of shedding of viable *Salmonella* after oral inoculation varies substantially between different *Salmonella* serovars. *Salmonella* Typhimurium vaccine strains show markedly higher intestinal colonization and excretion for longer duration than the same *Salmonella* Typhi vaccine strains [48]. The reduced fecal shedding of STMΔ*hfq* is an added advantage with respect to its impact on the environment.

An interesting observation was unlike the STM-WT, STMΔ*hfq* infection did not induce abortion in pregnant mice and the newly delivered pups remained healthy for several weeks. This observation hints at the safety of the usage of the vaccine strain in pregnant mice. Only subunit, killed or toxoid vaccines have been used so far to vaccinate pregnant animals instead of live attenuated vaccines due to the possibility of transmission of disease to the fetus. Moreover, these vaccines do not elicit any mucosal immunity. Abortions in pregnant animals in live stock due to *Salmonella* serovars cause immense economic losses [33]. Thus the use of *hfq* deletion mutant to vaccinate live stock can be considered.

To investigate the basis of protective immunity imparted by the STMΔ*hfq,* we examined the splenic T cell population. There was an increase in the total splenic CD4^+^ cells in STMΔ*hfq* infected mice compared to the naive as well as the STM-WT infected mice 7 days post infection. CD4^+^ T cells have profound effect on protective immunity as they assist the B-cells to produce more antibodies and in the generation of *Salmonella* specific CD8^+^ T cells [49], [50].We also observed that the numbers of CD4^+^ T cells were slightly reduced with the STM-WT infection. The reduction in the number of T cells by virulent *Salmonella* Typhimurium could be due to induction of apoptosis in a SPI-2 dependent manner by live bacteria [40], [51]. This may not be the case with the STMΔ*hfq* as SPI-2 encoded type three secretion system is down regulated [27], [28], [42]. In our study we did not observe any increase in the number of CD8^+^ T cells in an early stage of infection since *Salmonella* delays substantially the CD8^+^ T cell response until the second week of infection [52]. Further studies are needed in an *in vivo* model of *Salmonella* antigen presentation to ascertain the nature and specificity of the increased T cells.

It has been confirmed that the Th1 response (IFN-γ and TNF-α) is essential in inhibiting the systemic spread of *Salmonella* during initial stages of infection. However, infection with certain attenuated *Salmonella* strains can induce Th2 response [37], [53]. IFN-γ production by large number of *Salmonella* specific T cells and NK cells is at least in part responsible for the increased immunity against secondary infections. The levels of IFN-γ and IL-6 have been reported to be directly proportional to the severity of infection in *S.* Dublin infected mice [54]. This data supported our findings where we observed decrease in the level of IFN-γ and IL-6 in vaccinated and challenged mice. Nonetheless, the observed level could effectively clear the WT. The WT infected mice showed higher cytokine levels which could be attributed to the high virulence and burden of the wild type strain[55], [56], whereas vaccination with subsequent challenge showed lower cytokine levels, which could possibly be due to lower WT load on organs after vaccination [35], [56]. Apparently, an optimal cytokine level, which is neither as high as that in WT infected condition nor as low as that in the vaccinated alone, is sufficient to confer protective immunity.

Infection of B-cell deficient animals with attenuated *Salmonella* indicated the contribution of antibody mediated immune responses in protection against *Salmonella* infection [57]. In humans, high *Salmonella* Typhi Vi antigen specific antibodies correlate with the protection against infection. These two findings suggest that humoral immune response is important in effective clearance of primary *Salmonella* infection. In addition to LPS specific antibodies, OMP specific antibodies also contribute to the acquired resistance to *Salmonella* infection though to a lesser extent. Immunization of mice with purified OMPs can protect the mice from subsequent *Salmonella* infections and induces sustained lifelong memory responses without any added adjuvant [58], [59]. Cristina Gil-cruz et al. showed that antibodies to OmpD alone can reduce non-typhoidal *Salmonella* infection [60]. Moreover porins can act as activators of both innate and adaptive immune system. Absence of *hfq* leads to the chronic activation of the RpoE mediated envelope stress response and also markedly increases the OmpD levels [28]. In the present study, we observed that vaccination with STMΔ*hfq* increases the LPS and OMP specific IgG antibodies in the serum as well as S-IgA in the intestinal washes. As shown by Michetti et al., it can be inferred that the induced S-IgA would presumably block the entry of *Salmonella* at the mucosal surface possibly by immune exclusion [61]. Increased serum IgG can enhance the Fc-receptor and complement receptor mediated uptake of *Salmonella* by the phagocytic cells and bacterial clearance from blood by opsonization.

STMΔ*hfq* induced protective immunity can also be explained by enhanced antigen presentation of *hfq* deletion mutant by DCs. Cheminay et al. reported that virulent *Salmonella* inhibits the MHC-II dependent antigen presentation by dendritic cells and thereby decrease the activation of adaptive immune system. This capacity to inhibit the antigen presentation depends on the ability of the pathogen to alter the trafficking or maturation of the *Salmonella* containing vacuole (SCV) inside the DC which in turn depends on the presence of functional SPI-2 and production of nitric oxide (NO) [40]. The reason for increased presentation of STMΔ*hfq* vaccine strain could be due to lack of functional SPI-2 [27], [28], [42] or decreased production of nitric oxide (NO).

Hfq is required for the efficient translation of both stationary-phase sigma factor RpoS (σ^S^) and envelope stress response sigma factor RpoE (σE) [62]. Coynault et al. reported that the *rpoS* mutants are impaired in their capacity to colonize Peyer's patches after oral infection and are able to protect susceptible BALB/c mice against subsequent oral challenge with virulent STM-WT strain [16]. Vaccines with reduced ability to persist *in vivo* along with good immunogenicity are suitable for a human vaccine where side effects of vaccination are major concern. While our work was in progress, Karasova et al. reported that the *S.* Enteritidis *hfq* deletion mutant was susceptible to the components of the innate immune system such as serum and polymyxin B [63].

In summary, our present work demonstrates that *Salmonella* Typhimurium *hfq* deletion mutant can be a live attenuated vaccine, when given orally in a single dose can induce protective immune response upon subsequent challenge with virulent *Salmonella*. Not all attenuated *Salmonellae* strains can be presented well and this has been a stumbling block in the generation of a successful vaccine against *Salmonella*. Vaccination with *hfq* deletion mutant results in the active and specific protective immunity. Our work strongly suggests that the *hfq* deletion mutant can be considered for vaccination of humans and economically important livestock. Also the potential use of *hfq* deletion mutant as a heterologous antigen delivery system appears to be a very attractive option which still remains unexplored.

## Materials and Methods

### Ethics Statement

All the work with animals has been done with Institution approved ethics protocol by the Center for Animal facility Ethics Committee. The ethics number being CAF/ETHICS/189/2010. The ethics committee consisted of Institutional Animal ethics committee comprising of Animal facility Chairman, Convener and Scientists.

### Bacterial strains and growth conditions

*Salmonella* Typhimurium 14028 used in this study as the wild type parental strain was a kind gift from Prof. Michael Hensel (Max von Pettenkofer-Institute for Hygiene and Medizinische Mikrobiologie, Germany). The Δ*hfq* deletion mutant was constructed in the parental wild type strain. Bacteria were grown at 37°C in Luria Broth (LB) in the presence of carbencillin (50 µg/ml) or kanamycin (50 µg/ml) as and when required for the selection of the different strains.

### Eukaryotic cell lines and growth conditions

RAW 264.7 cells were a kind gift from Prof. Anjali Karande (Department of Biochemistry, Indian Institute of Science, India). INT-407, HT-29, HEK-293 and CaCo-2 cells were obtained from National Center for Cell Science (NCCS), Pune, India. All the cell lines were grown in Dulbecco's modified Eagle's medium (DMEM; Sigma), supplemented with 10% fetal calf serum (Sigma). For growth of CaCo-2 cells, DMEM was further supplemented with 1X non- essential amino acid solution. All the cells were maintained at 37°C in 5% carbon dioxide.

### Generation of the *hfq* deletion mutant in *Salmonella* Typhimurium

The *hfq* mutant was generated by one step gene inactivation strategy as described by Dastenko and Wanner [30]. Briefly, the STM 14028 strain was transformed with pKD46 carrying lambda red recombinase system under the arabinose inducible promoter. The transformants carrying the helper plasmid pKD46 were grown in LB with ampicillin (50 µg/ml) and 10 mM L-arabinose (Sigma) till the OD reached 0.38–0.4 at 600 nm. Electro-competent cells were prepared by washing the cells thrice with ice cold MilliQ water and 10% glycerol. PCR products containing the kanamycin-resistance gene (from pKD4) flanked by sequences upstream and downstream of the *hfq* gene was amplified with primers (HK1and HK2) and electroporated into the STM 14028 strain carrying pKD46. Electroporation was done according to the manufacturer's instructions (Bio-Rad). Mutant was selected for its ability to grow on LB containing kanamycin and the same was confirmed by confirmatory primers (HC1 and HC2) designed against the flanking loci of the *hfq* gene and internal primers (HI) designed against the kanamycin cassette sequence. In the STM-WT strain, a 310 bp band was amplified whereas in the STMΔ*hfq* strain, 1.5 Kb band (corresponding to Kanamycin cassette) was amplified. The deletion mutant thus generated was named STMΔ*hfq*.

Sequences of the primers used in this study are as follows:

HK1: 5′ atggctaaggggcaatctttacaagatccgttcctggtgtaggctggagctgcttcg 3′

HK2: 5′ttattcagtctcttcgctgtcctgttgcgcagtagcatatgaatatcctcctta 3′

HC1: 5′gcatataaggaaaagaga3′

HC2: 5′gataaacagcgcgtgaac3′

HI: 5′cagaccgttcagctggat3′ (used as reverse primer)

### Construction of *hfq* complemented strain

Genomic DNA isolated from *Salmonella* Typhimurium 14028 strain was used as template to amplify the *hfq* gene using the gene specific primers (Forward: 5′taggatccatggctaagg3′ and Reverse: 5′cgcaagcttttattcagt3′). The amplified product was purified and insert along with the purified vector pQE60 were digested with BamHI and Hind III. The digested vector and insert were ligated and transformed into *E. coli* DH5α competent cells and plated onto LA-carbencillin plates. The colonies were screened for plasmids having the appropriate insert by restriction digestion. The purified plasmid containing *hfq* gene was then transformed into STMΔ*hfq* electro-competent cells. The resulting strain was named as pQE60*hfq* strain.

### Intracellular proliferation assay

Different cell lines such as RAW 264.7, INT-407, HT-29 and CaCo-2 at a concentration of 1×10^5^ to 2×10^5^ per well were seeded in 24-well plates 12 h prior to infection. For the infection of RAW 264.7 cells, *S.* Typhimurium strains were grown till stationary phase in LB with the appropriate antibiotic. For infection of epithelial cells, stationary-phase cultures were diluted 1: 33 in LB and grown for 3 h (late-exponential phase) prior to infection to induce SPI-1 encoded genes required for invasion of non-phagocytic cells. The OD_600_ of the cultures was adjusted to 0.3 and the resulting bacterial suspensions were used to infect the cells at a multiplicity of infection (MOI) of about 10. The plates were centrifuged at 1000 rpm for 10 min and incubated at 37°C for 25 min. The cells were then washed with PBS to remove excess bacteria and fresh medium containing 100 µg/ml gentamicin was added. After 1 h, the medium was discarded followed by three PBS washes, and then medium containing 25 µg/ml gentamicin was added and incubated for further time periods. After specified incubation periods, cells were lysed with 0.1% Triton X-100 (Sigma) and were plated at different dilutions on LB agar containing appropriate antibiotic. The fold intracellular replication was calculated by dividing the intracellular bacterial load at 16 h by the intracellular bacterial load at 2 h.

### Animal infection

Six to eight weeks old BALB/c mice were issued from Central Animal Facility, Indian Institute of Science, Bangalore, India and maintained under specific pathogen free conditions. The bacterial strains were grown overnight at 37°C under shaking condition (180 rpm). Cells were centrifuged, washed, resuspended in sterile PBS and administered to mice at the indicated doses. Comparative analysis of the wild type versus vaccine strain burden in different organs was done by infecting mice orally with a dose of 10^7^ bacteria/mouse. After 4^th^ and 7^th^ day, mice were sacrificed followed by the isolation of liver, spleen and mesenteric lymph nodes. The organs were weighed and homogenized in 1ml PBS. The bacterial load in the organs was determined by plating serial dilutions of the homogenate on LB agar plates with selective antibiotic. The CFU was calculated per gram weight of the organ. Also, the total splenocytes were isolated for analyzing the CD4^+^ and CD8^+^ T-lymphocyte population.

### Safety of the vaccine strain

Safety of the vaccine strain in mouse model was determined by infecting the mice with a dose of 10^8^ bacteria/mouse (orally) and 10^4^ bacteria/mouse (intraperitoneally). The safety of the vaccine strain was analyzed in comparison with that of the wild type strain by observing the mice twice daily for mortality and morbidity. Same was tested in the pregnant mice which were infected (10^8^ bacteria/mouse) orally at mid pregnancy stage (12–14 days of gestation).

### Fecal shedding of the STM-WT and STMΔ*hfq* mutant

Cohorts of five mice each were orally inoculated either with STM-WT or STMΔ*hfq* mutant with a dose of 10^7^ bacteria. Fresh fecal pellets were collected after every 12 h till 4^th^ day and every 24 h from 4^th^ day onwards. To avoid drying of fecal pellets, mice were reared in cages with Whatmann papers. Freshly recovered fecal pellets were weighed and suspended in 1 ml of PBS. Samples were centrifuged at 1500 rpm for 10 min and plated onto plates with selective antibiotics. Data is represented after normalizing the CFU per gram weight of the fresh fecal pellets.

### LD_50_ determination

Six to Eight week old female BALB/c mice were fasted overnight prior to inoculation. To determine LD_50_ values, groups of ten animals were orally infected with specific inoculum for each strain. Animal deaths were recorded up to 3 weeks.

### Immunization of mice with STMΔ*hfq* mutant strain

#### Multiple booster dose immunization

Cohorts of five mice were orally primed with the STMΔ*hfq* mutant strain with a dose of 10^3^, 10^4^ or 10^5^ bacteria/mouse followed by two booster doses on 7^th^ and 14^th^ day. Control mice received PBS (Figure S2). All the subsequent steps (challenge, sacrifice) were carried out with a gap of seven days between them. After 7 days of second booster dose, the mice were challenged with 10^7^ STM-WT through oral route for analyzing organ infiltration. For survival assay, mice were challenged with 10^8^ CFU/mouse through oral route.

#### Single dose immunization

Mice were vaccinated with a single dose of STMΔ*hfq* (10^8^) and subsequently challenged with 10^7^ STM-WT on 7^th^ day of post vaccination. Mice were sacrificed 7 days post challenge and bacterial load in liver, spleen and mesenteric lymph nodes were determined as described above. For survival assay, mice were challenged through oral route with 10^8^ CFU of WT per mouse.

### Long term immunological memory induction by the STMΔ*hfq* vaccine strain

Cohort of five mice was vaccinated orally with a single dose of 10^8^ STMΔ*hfq* vaccine strain. After 52 days of post vaccination mice were challenged orally with 10^8^ STM-WT per mouse. To the control mice, PBS was administered. After challenging, vaccinated and non vaccinated mice were examined daily for morbidity and mortality.

### Analysis of serum cytokine levels

Blood was collected through cardiac puncture from the single dose immunized mice with or without challenge. Serum was separated and IFN-γ and IL-6 cytokine levels were assayed by ELISA (e-Bioscience) as per the manufacturer's protocol.

### Preparation of intestinal mucus

Mice were starved for 12 h before collecting intestinal mucus. Mice were scarified and intestinal region was collected and cut into small pieces. One PBS flush was given to remove fecal matter and intestine was reversed and mucus was collected by scrapping with forceps. The collected mucus was centrifuged at 12000 rpm and the collected supernatant was used for estimation of secretary IgA.

### Outer Membrane Protein Isolation (OMP)

Isolation of OMPs was carried out by a method of Hamid et al. [64]. Briefly, over-night grown bacterial culture was centrifuged and the pellet was washed thrice with 10 mM Tris-HCl (pH 7.5), and pellet equivalent to 1.0 g wet weight of bacteria was extracted with 20 ml of extraction buffer (10 mM Tris-HCl, pH 7.5, 10 mM EDTA, and 6 M urea) for 1 h at 4°C. The bacterial extract was dialyzed against MilliQ water for 3 days with frequent changes. The dialyzed material was centrifuged at 6000 rpm for 1 h. The supernatant containing surface proteins was collected and crude OMP sample was passed through a polymyxin B-agarose column (Sigma) to remove the contaminant endotoxin. Endotoxin levels in purified protein preparations was analysed using the E-Toxate kit (Sigma). The purified OMP sample was lyophilized and was stored at −20°C. The protein content in the samples was measured by Bradford method of protein estimation.

### Estimation of IgG and IgA antibodies from serum and intestinal mucus

LPS and OMP specific IgG and IgA antibodies in serum and intestinal mucus were assayed by ELISA. Purified LPS from *Salmonella* Typhimurium was obtained from Sigma-Aldrich where as OMPs were isolated as described below. Briefly, Nunc ELISA plate was coated with 100 ng/well of LPS or 500 ng/well of OMP in 100 µl of 0.02% Trichloro acetic acid (TCA). After 1 h incubation at 37°C, LPS or OMP coated wells were washed with wash buffer (0.5% PBS-Tween-20) and blocked with 3% BSA in the same. After 1 h incubation, wells were washed and incubated with dilutions of serum in wash buffer (1∶10) for 1 h at 37°C. Then wells were washed and 100 µl of anti-IgG and anti-IgA antibodies (Sigma) which are conjugated to HRP were added (1∶10,000 dilutions) followed by incubation at 37°C for 1 h and addition of 100 µl of substrate solution subsequently. After 15 min incubation, 50 µl of stop solution was added to each well and OD was measured at 450 nm. In case of intestinal mucus, equal amount of protein was taken after estimation by Bradford's method.

### Flow-cytometric analysis of T cell population

Splenocytes were isolated aseptically from and single-cell suspensions were obtained by crushing them between rough surface glass slides followed by erythrocyte lysis using RBC lysis buffer (0.1M NH_4_Cl, 1 mM KHCO_3_, 1 mM EDTA in H_2_O). Cells were stained separately with FITC-conjugated anti-CD4 (e-Biosciences) MAb, or PE-conjugated anti-CD8 (e-Biosciences) MAb. After 1 h incubation at RT, cells were washed with PBS and one million (10^6^) cells were taken for flow-cytometric analysis (BD FACS Canto-II). Data was analyzed with FACS Diva and Cell Quest Pro software (Becton Dickinson, Mountain View, Calif.).

### Purification of T lymphocytes

Five days of post infection of allogenic C57BL/6 mice with STM-WT, splenocytes were prepared by incubating small pieces of spleen in serum-free, calcium-free HBSS at 37°C for 45 min. This preparation was disaggregated by gentle pipetting to produce a single-cell suspension. The cells were then washed in HBSS, and erythrocytes were lysed with 1X RBC lysis buffer. Preparations were filtered to remove debris and were resuspended in RPMI with 10% FCS. Cells were incubated overnight to allow macrophages to adhere. Non adherent cells were transferred to fresh 30 mm dish which was coated with purified goat anti-mouse IgG antibody (20 µg/ml) and incubated for 6 h. After 6 h of incubation, non adherent cells were taken for assay. The purity of the T cells was checked by FACS using mouse anti-CD3 antibody conjugated to FITC (data not shown).

### Preparation and culture of Dendritic cells

Murine bone marrow derived dendritic cells were prepared from bone marrow of 6–8 week old naive BALB/c mice for antigen presentation assay as described by Cheminay et al [40]. Briefly, femurs were cut off at the ends and the marrow was flushed out. The cells were spun at 1,000 rpm for 10 min and resuspended in RPMI containing 10 ng/ml GM-CSF (Peprotech), 10% heat-inactivated FCS, 2 mM L-glutamine, 100 U/ml penicillin, 100 µg/ml streptomycin and cultured in 90 mm tissue culture dish. Finally, after 5 days of culture the medium was collected and the suspended cells were recovered and used for purification using CD11c magnetic beads. Further, the cells were found to be >90% CD11c^+^ by fluorescence-activated cell sorting. These cells were further checked for maturation markers like CD80 and CD86 and it was found that the purified DCs were immature (data not shown). The purified DCs were used for further experiments.

### *In vitro* antigen presentation assay

Modified method of Cheminay et al. was followed [40]. Briefly, dendritic cells were isolated from BALB/c as described earlier and were plated at a density of 5×10^4^ cells/well in 96-well flat-bottomed tissue culture plates. Bacterial infection of the DCs was performed as described in intracellular proliferation assay. Twelve hours post infection, purified splenic T cells from allogenic C57BL/6 mice were added to each well. DC and T cell ratio was maintained at 1∶10 and 1∶1. Cells were co-cultured for 72 h and then pulsed for 16 h with [^3^H] thymidine (0.1 µCi/well). Cells were harvested on glass fiber filters using a semi-automated cell harvester (Nunc, Denmark). [^3^H] thymidine was measured in a liquid scintillation counter (Rack Beta) and T-cell proliferation was plotted as mean counts/min of triplicate wells.

### Statistics

*In vitro* data were analyzed by Student's “t” test using Sigma Plot. Mann Whitney *U* test were performed for mouse experiment using commercially available software (Graph Pad Prism, San Diego, CA). The mortality and survival data were analyzed using Fishers exact test. Results were determined to be statistically significant when a P value of less than 0.05 was obtained.

## Supporting Information

Figure S1 Complementation of the STMΔ*hfq* strain with pQE60*hfq* restores virulence. Intracellular replication of STM-WT, STMΔ*hfq* and complemented strain in INT-407 (A) and RAW 264.7 (B) cell line. INT-407 and RAW 264.7 cell lines were infected with a MOI of 10 and lysed at 2 h and 16 h post infection. Bacterial fold replication was calculated from 2 to 16 h as shown in the graph. (C) Organ loads of STM-WT, STMΔ*hfq* and complemented strain. Three groups of mice (6 each) were infected with 10^7^ CFU/mouse orally with each strain separately and sacrificed on 4^th^ day of post infection. Bacterial counts in spleen, MLN and liver were measured by plating on respective antibiotic plates and were shown as CFU/gm.wt with standard errors. Graphs are representative of two independent experiments with similar results. Statistical significance was defined as follows: (*p<0.05; **p<0.005) (Student's *t* test & Mann-Whitney U test).
(TIF)

Figure S2 Immunization strategy followed to evaluate the vaccine potential of the STMΔ*hfq* deletion mutant (A) multiple immunization strategy; mice were primed with vaccine strain followed by two booster doses on 7^th^ and 14^th^ day and then challenged with virulent *Salmonella* strain on 7^th^ day after last booster dose. (B) Single dose of vaccination: mice were vaccinated and then challenged with virulent *Salmonella* strain after 7 days of post vaccination. For CFU analysis mice were challenged with 10^7^ and for survival assay 10^8^ CFU of WT.
(TIF)

Figure S3 Flow cytometric analysis of CD8^+^ T cell population in the spleen on 4^th^ day and 7^th^ day of post infection. Groups of mice were inoculated with the STM-WT or STMΔ*hfq* with dose of 10^7^ bacteria per mouse. Uninfected mice were used as control. Splenocytes were isolated on 4^th^ and 7^th^ day of post infection from both infected and control mice and stained with PE-conjugated anti-CD8 MAb. The relative levels of CD8^+^ T-lymphocytes were measured through FACS. Data was analyzed with BD Cell-Quest software and represented by dot plots. The results are representative of two independent experiments. Each group consisted of 4-5 mice.
(TIF)

Figure S4 Flow cytometric analysis of splenic CD8^+^ T cell population in vaccinated and unvaccinated mice with or without challenge. Group of mice were orally given PBS or 10^8^ STM Δ*hfq* and then challenged after seven days of post vaccination with 10^7^ CFU of STM-WT per mouse. On 7^th^ day post vaccination (from unchallenged mice) and 7^th^ day post challenge (from challenged mice) spleen were isolated and single cell suspension of splenocytes were prepared followed by staining with FITC-conjugated anti-CD8 MAb. The relative levels of CD8^+^ T-lymphocytes were measured through FACS. Data was analysed by BD Cell-Quest software and represented through dot plot. Each group consisted of 4-5 mice.
(TIF)

Figure S5 Estimation of the serum IgG and intestinal S-IgA levels 4 weeks after single dose of vaccination. Group of mice were orally given PBS or vaccine strain (10^8^), serum and intestinal mucus were collected 4 weeks post vaccination. Serum IgG (B&D) and intestinal S-IgA (A&C) antibodies specific for LPS and OMP were measured by ELISA. The samples were assayed in triplicate and the antibody titer is expressed as the absorbance at 450 nm. Result presented is one of two independent experiments. Statistical significance was defined as follows: (*p<0.05; ** p<0.005) (Student's *t* test). (n = 5-6).
(TIF)
