# Supplementary figures and images for: Salmonella enterica Serovar Typhimurium Lacking hfq Gene Confers Protective Immunity against Murine Typhoid
Source: PLoS One. 2011 Feb 9;6(2):e16667. doi: 10.1371/journal.pone.0016667 (PMC3036662; doi:10.1371/journal.pone.0016667)

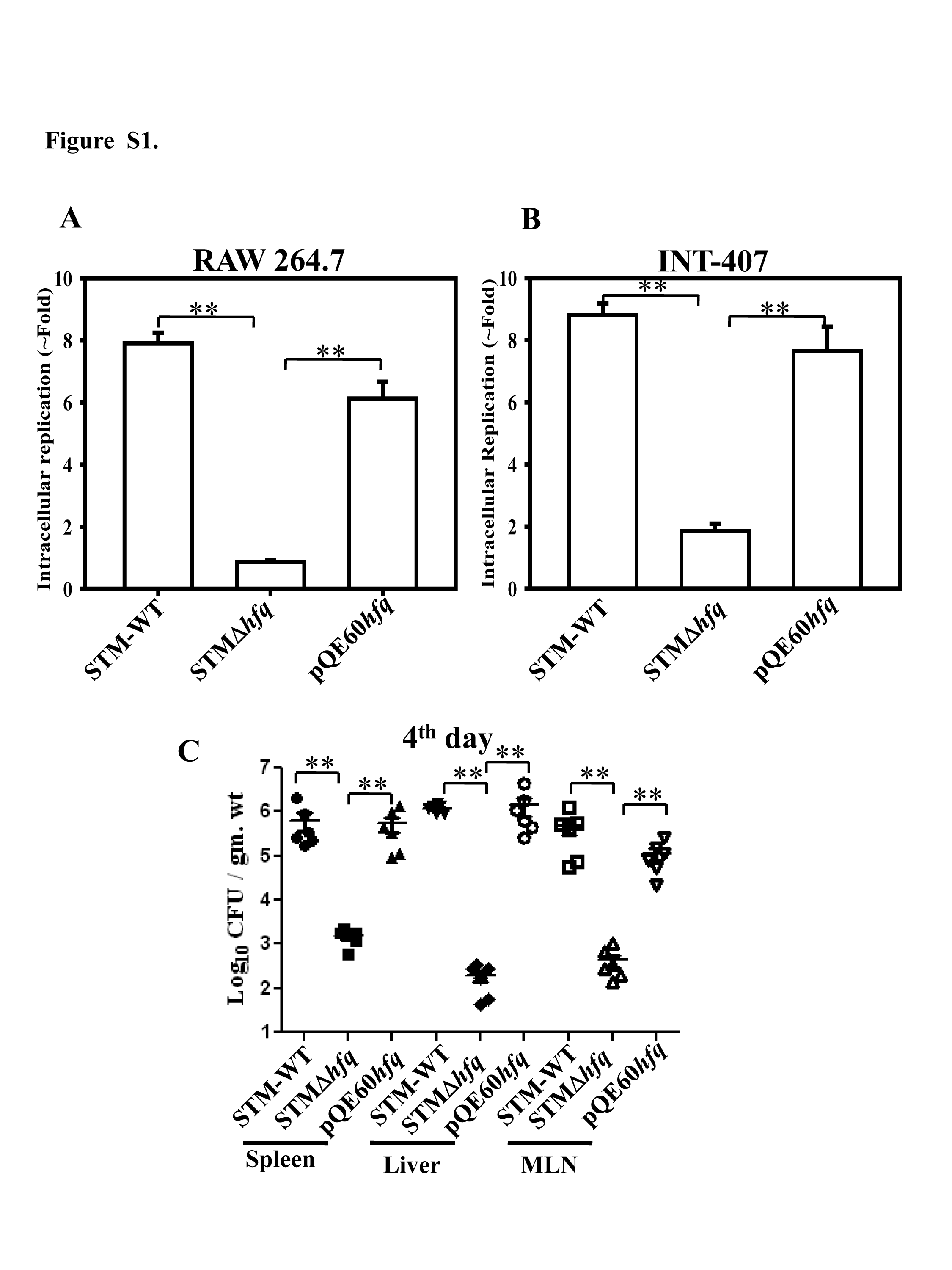

Supplement: Figure S1 — Complementation of the STMΔhfq strain with pQE60hfq restores virulence. Intracellular replication of STM-WT, STMΔhfq and complemented strain in INT-407 (A) and RAW 264.7 (B) cell line. INT-407 and RAW 264.7 cell lines were infected with a MOI of 10 and lysed at 2 h and 16 h post infection. Bacterial fold replication was calculated from 2 to 16 h as shown in the graph. (C) Organ loads of STM-WT, STMΔhfq and complemented strain. Three groups of mice (6 each) were infected with 107 CFU/mouse orally with each strain separately and sacrificed on 4th day of post infection. Bacterial counts in spleen, MLN and liver were measured by plating on respective antibiotic plates and were shown as CFU/gm.wt with standard errors. Graphs are representative of two independent experiments with similar results. Statistical significance was defined as follows: (*p<0.05; **p<0.005) (Student's t test & Mann-Whitney U test). (TIF) [file pone.0016667.s001.tif]

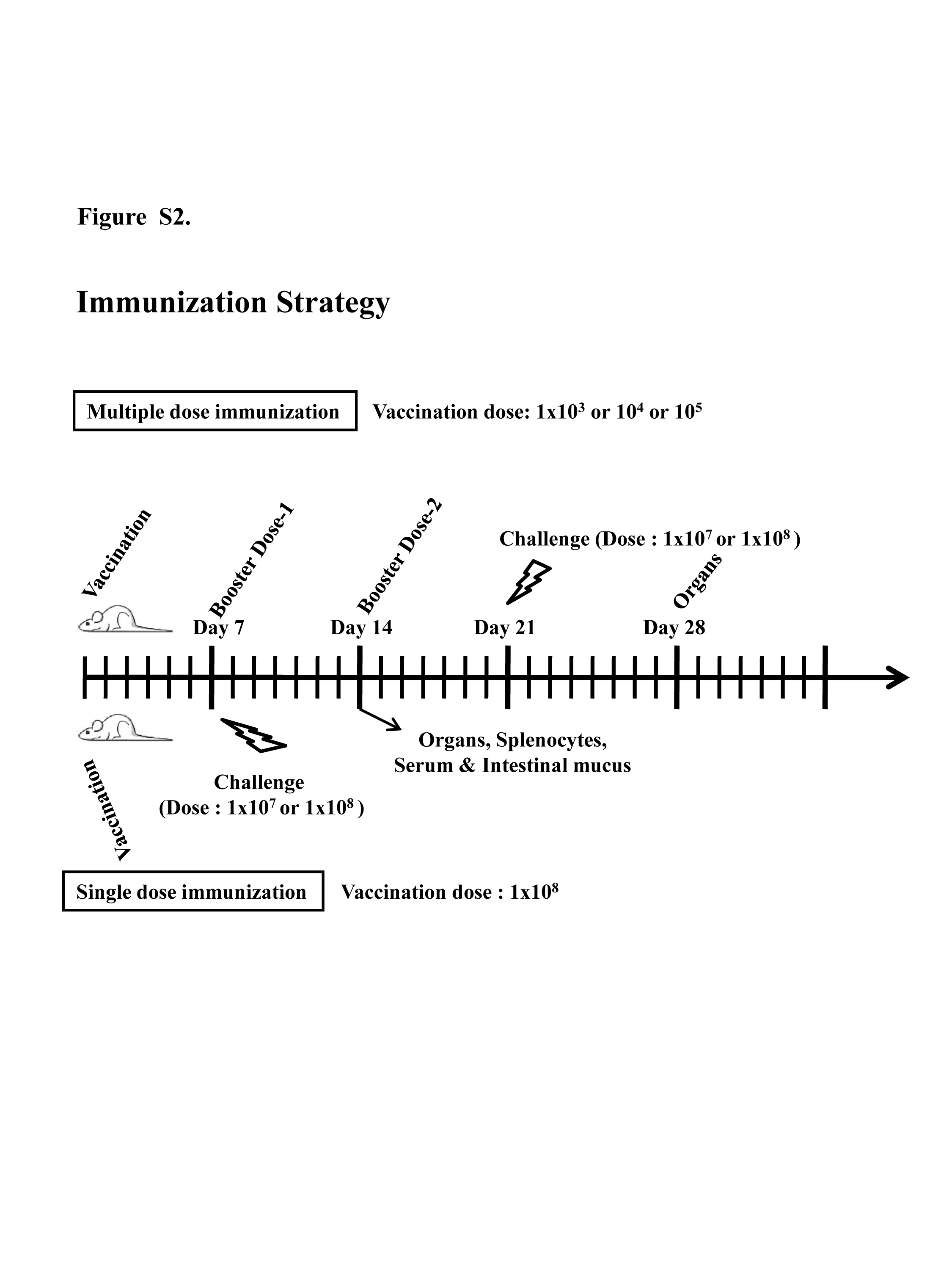

Supplement: Figure S2 — Immunization strategy followed to evaluate the vaccine potential of the STMΔhfq deletion mutant (A) multiple immunization strategy; mice were primed with vaccine strain followed by two booster doses on 7th and 14th day and then challenged with virulent Salmonella strain on 7th day after last booster dose. (B) Single dose of vaccination: mice were vaccinated and then challenged with virulent Salmonella strain after 7 days of post vaccination. For CFU analysis mice were challenged with 107 and for survival assay 108 CFU of WT. (TIF) [file pone.0016667.s002.tif]

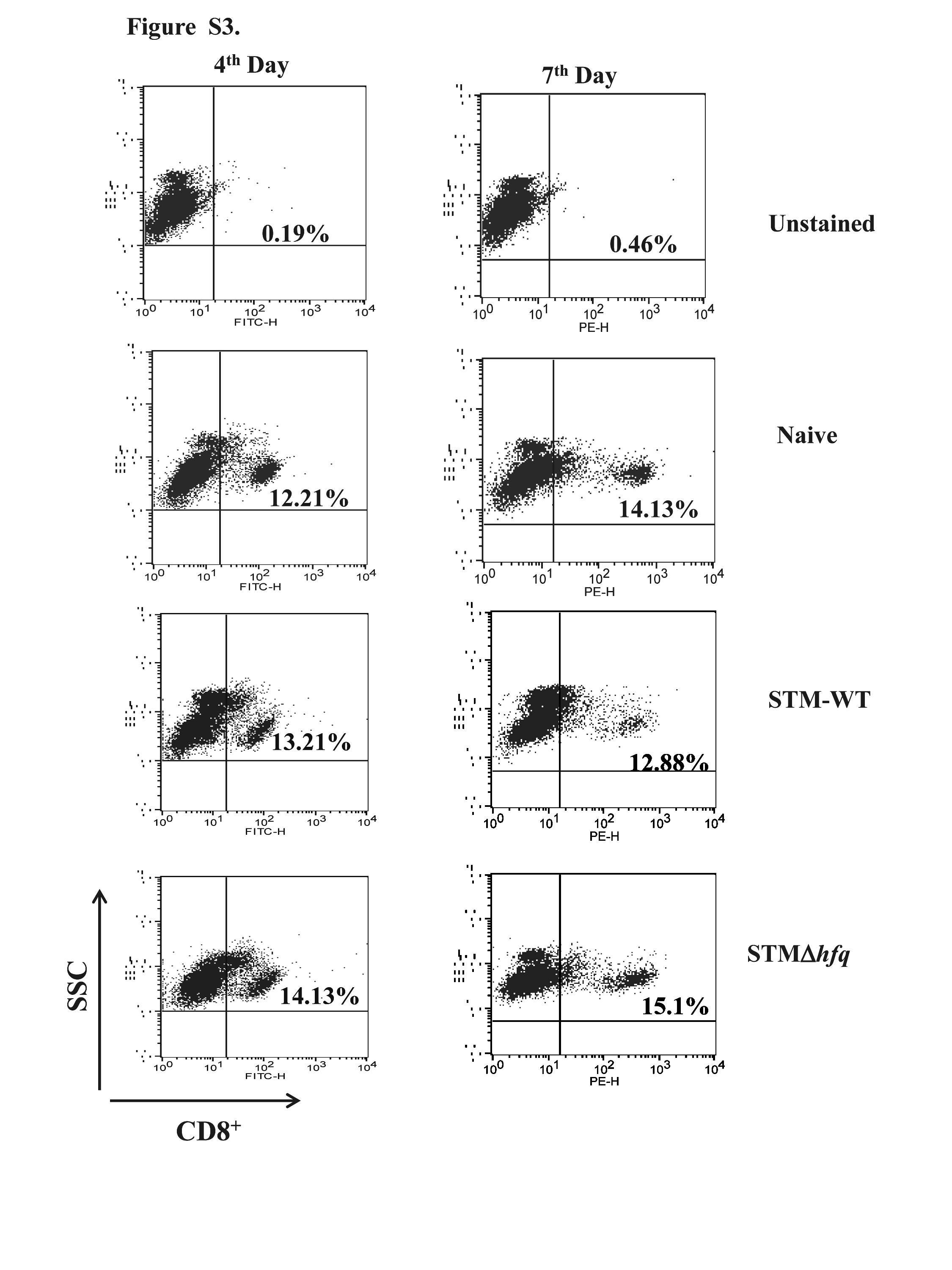

Supplement: Figure S3 — Flow cytometric analysis of CD8+ T cell population in the spleen on 4th day and 7th day of post infection. Groups of mice were inoculated with the STM-WT or STMΔhfq with dose of 107 bacteria per mouse. Uninfected mice were used as control. Splenocytes were isolated on 4th and 7th day of post infection from both infected and control mice and stained with PE-conjugated anti-CD8 MAb. The relative levels of CD8+ T-lymphocytes were measured through FACS. Data was analyzed with BD Cell-Quest software and represented by dot plots. The results are representative of two independent experiments. Each group consisted of 4-5 mice. (TIF) [file pone.0016667.s003.tif]

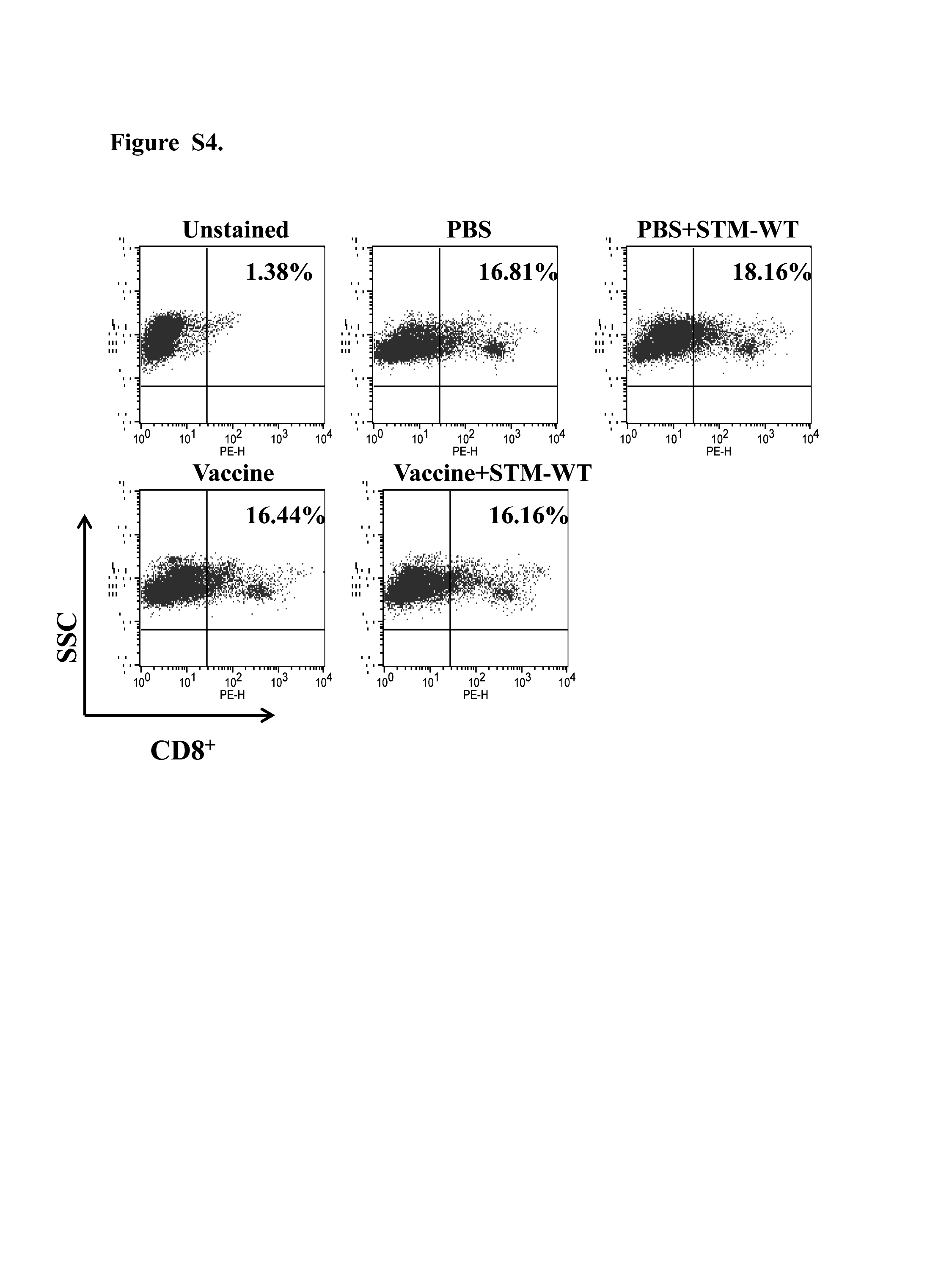

Supplement: Figure S4 — Flow cytometric analysis of splenic CD8+ T cell population in vaccinated and unvaccinated mice with or without challenge. Group of mice were orally given PBS or 108 STM Δhfq and then challenged after seven days of post vaccination with 107 CFU of STM-WT per mouse. On 7th day post vaccination (from unchallenged mice) and 7th day post challenge (from challenged mice) spleen were isolated and single cell suspension of splenocytes were prepared followed by staining with FITC-conjugated anti-CD8 MAb. The relative levels of CD8+ T-lymphocytes were measured through FACS. Data was analysed by BD Cell-Quest software and represented through dot plot. Each group consisted of 4-5 mice. (TIF) [file pone.0016667.s004.tif]

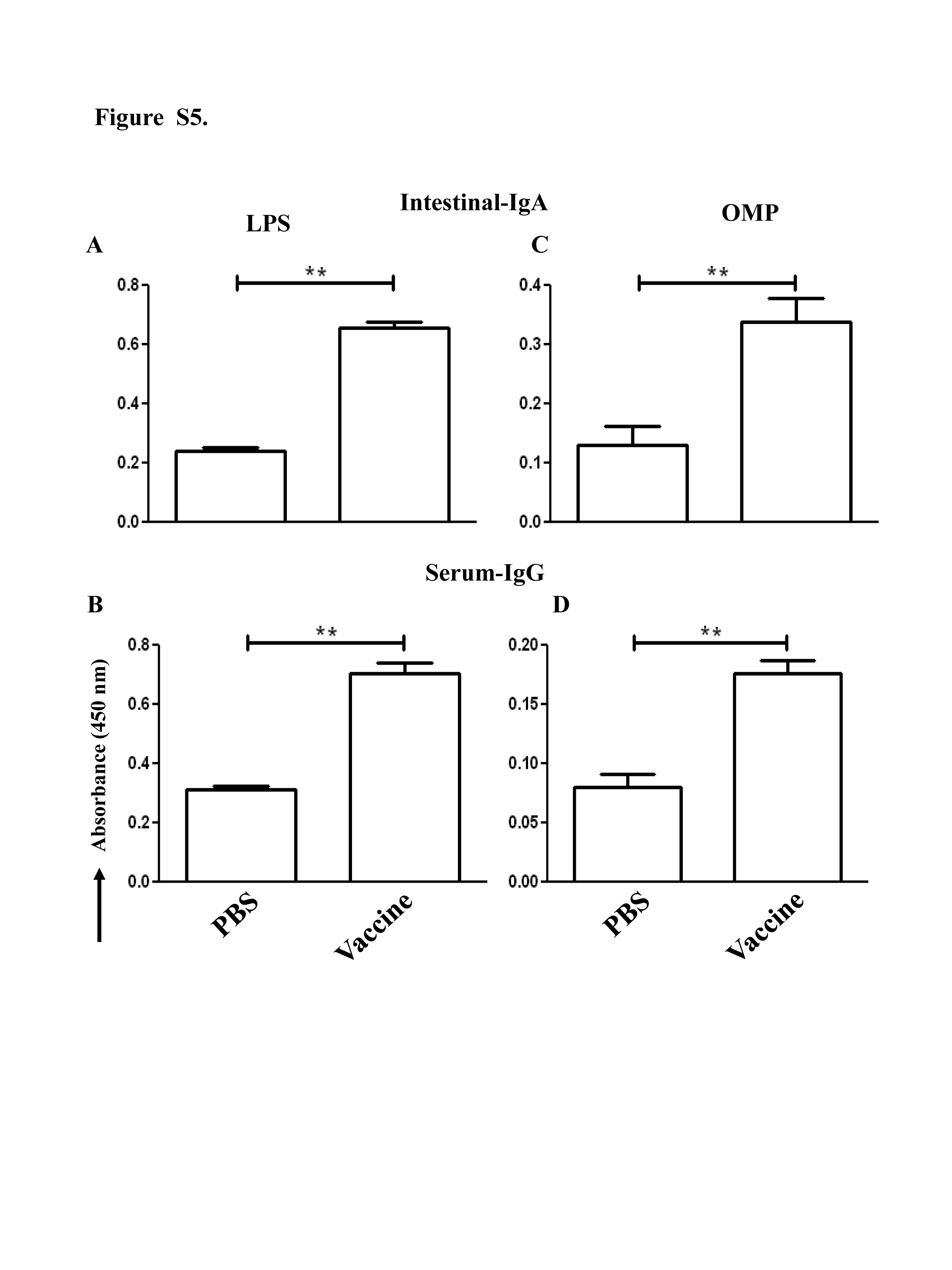

Supplement: Figure S5 — Estimation of the serum IgG and intestinal S-IgA levels 4 weeks after single dose of vaccination. Group of mice were orally given PBS or vaccine strain (108), serum and intestinal mucus were collected 4 weeks post vaccination. Serum IgG (B&D) and intestinal S-IgA (A&C) antibodies specific for LPS and OMP were measured by ELISA. The samples were assayed in triplicate and the antibody titer is expressed as the absorbance at 450 nm. Result presented is one of two independent experiments. Statistical significance was defined as follows: (*p<0.05; ** p<0.005) (Student's t test). (n = 5-6). (TIF) [file pone.0016667.s005.tif]
